# Tissue Proteases and Immune Responses: Influencing Factors of COVID-19 Severity and Mortality

**DOI:** 10.3390/pathogens9100817

**Published:** 2020-10-06

**Authors:** Natália Mulinari Turin de Oliveira, Isabella Fernandes da Silva Figueiredo, Liziane Cristine Malaquias da Silva, Karien Sauruk da Silva, Laryssa Regis Bueno, Bruna Barbosa da Luz, Cláudia Rita Corso, Maria Fernanda Paula Werner, Elizabeth Soares Fernandes, Daniele Maria-Ferreira

**Affiliations:** 1 Faculdades Pequeno Príncipe, Av. Iguaçu No 333, Curitiba, PR 80250-200, Brazil; natimulinari@gmail.com (N.M.T.d.O.); bellaafigueiredo@hotmail.com (I.F.d.S.F.); lizianemalaquias0@gmail.com (L.C.M.d.S.); kariensauruk@outlook.com (K.S.d.S.); laryssaregis@hotmail.com (L.R.B.); claudia_rcorso@hotmail.com (C.R.C.); elizabeth.fernandes@pelepequenoprincipe.org.br (E.S.F.); 2 Instituto de Pesquisa Pelé Pequeno Príncipe, Av. Silva Jardim No 1532, Curitiba, PR 80250-200, Brazil; 3 Departamento de Farmacologia, Universidade Federal do Paraná, Curitiba, PR 81531-980, Brazil; brunabarbosaluz@gmail.com (B.B.d.L.); mfernanda.werner@ufpr.br (M.F.P.W.)

**Keywords:** COVID-19, SARS-CoV-2, host factors, tissue proteases, immune responses, disease outcome

## Abstract

The coronavirus disease 19 (COVID-19) is caused by the highly transmissible severe acute respiratory syndrome coronavirus 2 (SARS-CoV-2), which has affected the global population despite socioeconomic status and amazed surveillance agencies for its incidence, mortality, and recovery rates. COVID-19 affects all age groups; however, it is suggested to progress into severe disease and cause mortality in over 10% of the confirmed cases, depending on the individual characteristics of the affected population. One of the biggest unanswered questions it is why only some individuals develop into the severe stages of the disease. Current data indicate that most of the critically ill are the elderly or those with comorbidities such as hypertension, diabetes, and asthma. However, it has been noted that, in some populations, severe disease is mostly observed in much younger individuals (<60-years old) with no reported underlying medical conditions. Certainly, many factors may contribute to disease severity including intrinsic host factors such as genetic variants, the expression levels of tissue proteins, among others. Considering all these aspects, this review aims to discuss how the expression levels of tissue proteases and the different profiles of immune responses influence the susceptibility to COVID-19 as well as disease severity and outcome.

## 1. Introduction

The emerging ongoing outbreak of pneumonia known as coronavirus disease 19 (COVID-19) is caused by severe acute respiratory syndrome coronavirus 2 (SARS-CoV-2) and has posed as a massive global public health crisis that rapidly disseminated worldwide. As of 9 September 2020, more than 27 million cases of the disease have been globally recorded, with nearly 900,000 deaths, exceeding the combined number of the individuals infected by the Middle East respiratory coronavirus (MERS-CoV) and respiratory syndrome coronavirus SARS-CoV, as well as their associated deaths [1].

Coronaviruses (CoVs) belong to the family Coronaviridae, order Nidovirales, and are the largest known RNA viruses classified into the following four types: α, β, γ, and δ [2]. They have long been recognized as important pathogens that can be transmitted from human to human causing mild to severe respiratory tract and intestinal infections with high morbidity and mortality [3]. SARS-CoV-2 belongs to subgenus *Sarbecovirus* and comprises a large β-enveloped, non-segmented positive-sense RNA virus, with approximately 30,000 bases in length [4] encoding 9860 amino acids [5]. Full-length genome sequences indicate that SARS-CoV-2 is closely related to SARS-CoV sharing 79.6% sequence identity [6]. Like other CoVs, SARS-CoV-2 is composed of four structural proteins, known as S (spike), E (envelope), and M (membrane) proteins, which compose the viral envelope, and N (nucleocapsid) protein, which holds the RNA genome [5]. The S protein recognizes and binds to its receptor, the angiotensin-converting enzyme 2 (ACE2), is a vital pathway for virus entry and replication into the human lung alveolar epithelial cells [4].

Although most of the infected individuals are asymptomatic or display mild symptoms, COVID-19-associated severe cases are characterized by progressive respiratory failure and lethal pneumonia. Current reports suggest that severe cases of COVID-19 are most frequent in the elderly population or those with comorbidities, including asthma, heart disease, and diabetes [6]. In contrast, in some specific populations, severe cases have been also often observed in much younger individuals (<60 years old) with no disabling conditions [7]. The broad range of individuals affected by severe disease has raised a highly important debate on the importance of host–pathogen interactions as major influencing factors of disease outcome.

By comprehending these variables, it will be easier to identify which populations are to suffer the hardest with COVID-19, and tailor better management rules and treatment options to avoiding disease contagion and progression. Herein, we discuss some of the intrinsic factors associated with COVID-19 severity and mortality, with a special focus on the tissue proteins and immune responses involved in SARS-CoV-2 infection.

## 2. Pathogenesis of COVID-19

An elevated case fatality rate in males was previously observed for respiratory tract infections such as the ones caused by SARS-CoV and MERS-CoV [8,9]. A similar gender-related influence on COVID-19 has also been suggested, with men more prone to severe disease and mortality. Indeed, SARS-CoV-2 mostly infected males in Wuhan (68%) [10]. Additionally, in Italy, 58% of the diagnosed COVID-19 patients are males, with the highest mortality rates observed for this gender (70%) [11]. Several factors are suggested to underlie the gender influence in COVID-19 including the sex hormones, immunity, ACE2 activity, as well as behavioral and cultural habits, such as smoking [12]. In fact, male smokers in China represent 288 million individuals in contrast to the 12.6 million of declared female smokers [13]. A similar scenario is observed in Italy. Smoking prevalence is much higher among men than women at any age group [14].

Another important aspect to be considered is the aging of the population, as it has been pointed out as a risk factor for severe disease and mortality, as well as for increased contamination. Notably, recent reports have shown that the elderly are the most susceptible to lethality in countries such as Italy and China, which present high numbers of individuals over 60 years old [15]. Indeed, people ranging from 40 to 49 years of age had an estimated case fatality rate of 0.4%, whereas individuals ≥ 80 years old presented 20.2% mortality [16]. A worse prognosis was also strongly associated with underlying comorbidities [17], with case fatality rates higher in individuals with cardiovascular and chronic respiratory diseases, diabetes, and tumors [18]. However, rare severe cases and mortality have also been observed in children, with reports associating them with the development of a hyperinflammatory shock [19]. In some countries, a significant proportion of previously healthy individuals ≤ 50 years of age have also developed severe COVID-19, with fatalities being registered in this group [20]. Although surprising, statistics on the subject are yet scarce as data continue to be collected from all around the world with this ongoing disease.

These findings suggest that not only age and gender, but also other intrinsic host factors are essential to predisposing an individual to experiencing severe disease and lethality. In this context, and in the hope of defining better options for the clinical management of COVID-19, ongoing research studies have been dedicated to dissecting the genetic characteristics as well as the gene and protein expression patterns of human cells that may influence disease outcome.

**Figure 1:**
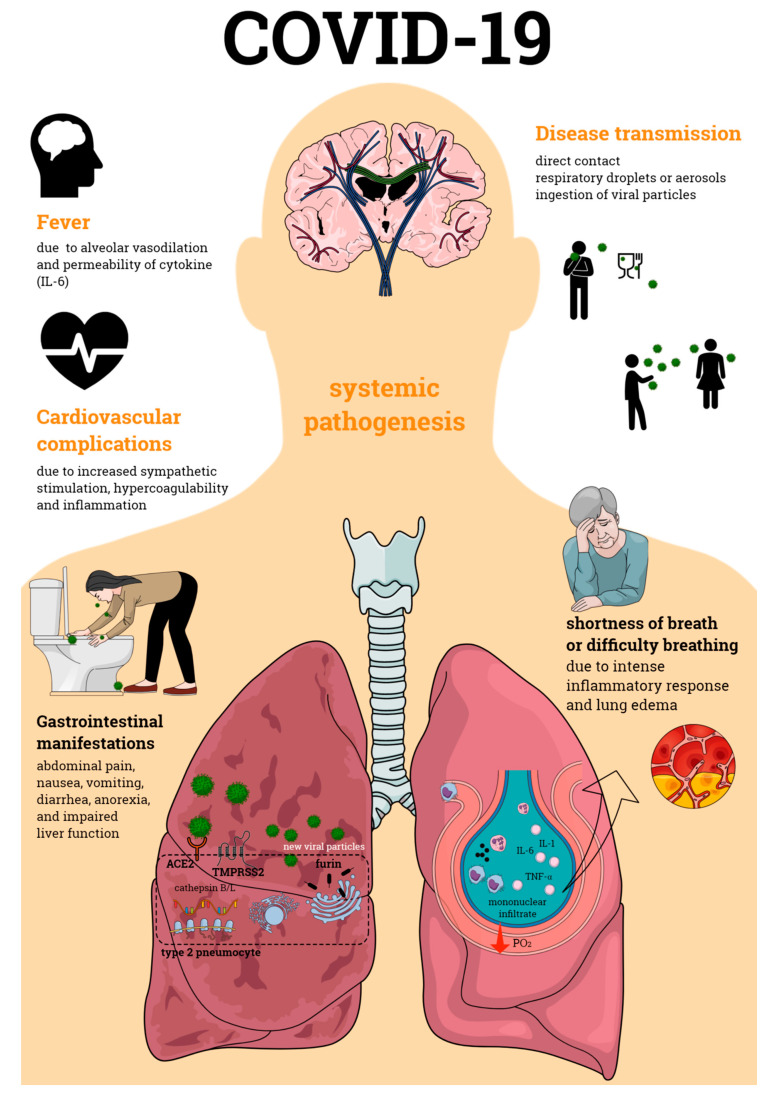
Coronavirus disease 19 (COVID-19) pathogenesis. COVID-19 is an infectious disease caused by the severe acute respiratory syndrome coronavirus 2 (SARS-CoV-2), which primarily affects the respiratory tract causing bilateral pneumonia. Its transmission may occur through direct contact and respiratory droplets or aerosols, and through the ingestion of viral particles. COVID-19 also affects multiple organs, often leading to organ failure and death of the individual affected by the infection. Organic complications observed during the infectious process include those seen in the lungs, cardiovascular and gastrointestinal systems, among others. All of these contribute to the enormous morbidity and mortality of SARS-CoV-2 infection.

Primarily defined as a disease of the respiratory tract, SARS-CoV-2 infection is now known to cause a systemic pathogenesis, targeting multiple organs and ultimately leading to their failure and to patient death (Figure 1). Disease transmission is still uncertain, but it is thought to happen through direct contact, respiratory droplets, or aerosols, and even by ingestion of viral particles [21]. Once transmitted to an individual, the virus enters the human cells via a direct interaction of its spike protein with the host ACE2. ACE2 is highly expressed not only in human alveolar epithelial cells, but also in various others including the tongue and oral mucosa epithelial cells, leukocytes, blood vessels, heart, kidney, endothelium, and intestine [22,23,24]. Other broadly expressed host proteins contribute to an effective infection of SARS-CoV-2 into human cells including the proteases furin and furin-like proteins [25], transmembrane protease serine 2 (TMPRSS2), and the cathepsin B and L (CatB/L) [26]. At this early phase of infection, the expression profiles of these proteins as well as interferon (IFN) production are determinant pathways for disease outcome. As disease progresses, cellular immune responses become essential players in the host fight to SARS-CoV-2. Imbalances in these pathways such as defective IFN production or ineffective cell migration/activation may, therefore, result in a worsened disease prognosis.

Different studies have investigated the immune responses involved in COVID-19; however, few of them have been able to correlate disease severity and the different players of immunity. So far, differences in inflammatory mediator release and immune cells have been observed. Following lung infection, there is an intense inflammatory response with the lungs exhibiting edema and mononuclear infiltrates, especially of lymphocytes [27]. At the systemic level, lymphopenia has been observed and linked to an increased severity of COVID-19, in addition to mortality [28]. Individuals with severe disease also present higher neutrophil-to-lymphocyte ratios, low numbers of CD4^+^, and deficiency of memory and regulatory T cells [29]. In another report, a patient with mild to moderate disease exhibited normal lymphocyte and neutrophil counts; however, T lymphocytes (CD8^+^ and CD4^+^ cells) were elevated at one-week post-symptom initiation [30]. More recently, neutrophil-derived extracellular traps (NETs) were associated with severe COVID-19 [31]. Of note, NETs are involved in both inflammation and thrombosis, this later considered as another hallmark of SARS-CoV-2 infection [32]. Systemic inflammatory levels were also associated with disease severity. Indeed, while high levels of interleukin (IL)-6 and IL-10 were detected in severe disease, low levels of cytokine and chemokines were found in patients with milder infection [29,30]. Increased circulating levels of IL-6 were also correlated with cardiac injury and mortality in COVID-19 patients [33]. Interestingly, IFN-mediated responses were suggested to be delayed by SARS-CoV-2 [34]; however, the bronchoalveolar release of IFN as well as the expression of IFN-associated genes seem to correlate with severe disease in some critically ill individuals [35].

Cardiovascular complications such as coronary heart disease, heart failure, and cerebrovascular disease have been observed in COVID-19 patients as a result of infection-related myocarditis and/or ischemia. Increased sympathetic stimulation, hypercoagulability, and inflammation are thought to contribute to these cardiovascular events [36,37,38]. Another remarkable complication of SARS-CoV-2 infection is renal failure, which has been frequently observed in patients with the severe form of COVID-19 [39,40,41]; this may be either a direct effect of the virus, or a result from the hypoxia observed following infection. Additionally, pre-existing cardiovascular and kidney diseases have been considered as risk factors for COVID-19; therefore, it is possible that infection can contribute to exacerbate such conditions.

Although less common than the cardiovascular and kidney alterations, gastrointestinal tract manifestations have also been implicated in COVID-19. Abdominal pain, anorexia, impaired liver function, diarrhea, nausea, and vomiting have been noted in SARS-CoV-2 infection and were more frequent in individuals in critical condition [40,42,43]. Post-mortem analysis indicated that patients who died of severe disease presented small intestine alterations such as dilatation and stenosis; gastrointestinal mucosal degeneration, necrosis, and shedding; in addition to edema and lymphocyte influx into the esophagus, stomach, duodenum, and rectum lamina propria [44,45]. It is estimated that over 30% of COVID-19-positive patients present viral load in their stool samples, even when their nasopharyngeal samples are negative for SARS-CoV-2 [46,47,48]. Furthermore, viral proteins were found in gastric, duodenal, and rectal epithelial cells [45]. This later finding suggests that the gastrointestinal symptoms of COVID-19 may result from the direct interaction of SARS-CoV-2 with the gastrointestinal cells.

Testicular tissue damage and defects in spermatogenesis were previously observed during the SARS-CoV outbreak [27]. Despite the scarce literature, recent reports also indicate that the SARS-CoV-2 infection may affect the testicular tissue. Adult human scRNA-seq datasets suggest that the testis is potentially vulnerable to SARS-CoV-2 infection [49]. It is important to highlight that these are early findings, and it is currently uncertain how COVID-19 affects the reproductive functions over the short and long terms. Therefore, monitoring of male patients following recovery from acute infection is strongly advised.

Collectively, these evidences indicate that both the inflammatory scenario and the ubiquitous expression of ACE2 and the aforementioned tissue proteases contribute to the variety of symptoms of COVID-19 infection, which range from dyspnea and bilateral pneumonia, to tachycardia and diarrhea.

## 3. Illness Degree and Association with Host Tissue Proteins and Immune Responses

An important question on the current pandemic scenario is why not all infected individuals develop life-threatening disease. Although most of the critical cases are related to the elderly or those with underlying problems, some individuals are asymptomatic or display mild flu-like symptoms. Certainly, the susceptibility to infection cannot be attributed only to demographic or socioeconomic factors, as genetic differences may also strongly account for COVID-19 outcome. Genetic variability of genes involved in the expression of host–pathogen interaction proteins and inflammatory mediators are among the suggested host predisposing factors that may influence disease severity and mortality. This is discussed below.

### 3.1. Tissue Protein Expression

Among the proteins suggested to contribute to cell infection by SARS-CoV-2, ACE2 is perhaps the most well investigated. As previously discussed, SARS-CoV-2 invasion and trafficking into human cells are primarily driven by the interaction of its spike protein with human ACE2 [26]. Some reports have suggested that variations in ACE2 gene sequences may influence cell infection and viral load and, consequently, disease severity or resistance to SARS-CoV-2 (Table 1). In 2020, by using molecular modeling tools, Hussein et al. [50] identified ACE2 encoding variants that corresponded to the binding sites for the SARS-COV-2 spike protein. They found that most of the ACE2 variants present similar binding affinities to the SARS-CoV-2 spike protein. However, two ACE2 alleles, namely rs143936283 (E329G) and rs73635825 (S19P), displayed low binding affinity to the virus and lacked key residues important to the complex formation with the SARS-CoV-2 spike protein.

Additional studies on ACE2 tissue expression and distribution were also performed. ScRNA-seq analysis revealed that Asian males present higher lung expression of ACE2 in comparison to Caucasian and African American individuals [23]. Moreover, the frequency of allele variants associated with increased ACE2 tissue expression was found to be higher in East Asian populations in comparison to other populations, such as the European, African, South Asian, and American ones [51]. In contrast, no substantial evidences have associated ACE2 expression with COVID-19 severity [13,53]. However, more robust studies on large cohorts need to be performed in order to establish whether there are any direct links between ACE2 expression and disease outcome. Interestingly, tobacco smoking was found to increase the pulmonary expression of ACE2, including in the small airway epithelia [61,62]. These findings suggest that smokers may be more susceptible to COVID-19.

An important aspect to consider is the percentage of individuals with diseases such as hypertension and diabetes who developed severe COVID-19. ACE2 is essential not only to virus entry, but also to the renin–angiotensin–aldosterone system, exerting a protective role in cardiovascular diseases [63] and diabetes [64]. So far, the association between ACE2 expression and the susceptibility to SARS-CoV-2 infection is conflicting. In fact, while some data suggest that an increased expression of ACE2 favors the progression of the disease [52], others indicate that COVID-19 leads to a reduction of ACE2 activity in elderly individuals with chronic diseases, resulting in angiotensin II/ACE2 regulation imbalance and loss of ACE2 protective effects [54]. Furthermore, the low incidence of severe cases in children may be related to lower ACE2 expression [65,66]. Of importance, it has also been speculated on whether treatment with ACE inhibitors or angiotensin receptor blockers affect the course of COVID-19 as they may enhance the disruption of the angiotensin II–ACE2 axis [67].

Alterations in the expression patterns of host tissue proteases necessary to virus replication may also favor resistance or susceptibility to COVID-19. In this context, genetic variants of TMPRSS2 have been investigated (Table 1). Analysis of the Italian exome and GnomAD data detected genetic variants that may impact TMPRSS2 expression and its catalytic activity. The Italian population presented a decreased burden of deleterious variants compared with other European populations [53]. It is thus possible that Italians have enhanced TMPRSS2 activity, and that this results in a higher risk of a more severe form of COVID-19 [53]. Notably, genetic variants for TMPRSS2 were already shown to enhance the risk to severe A(H1N1) pdm09 and A(H7N9) influenza [54]. Interestingly, as suggested for ACE2, the low expression of TMPRSS2 in the airway epithelial and alveolar type 2 cells of infants and young children may protect them from SARS-CoV-2 infection [66]. Despite the early findings relating ACE2 and TMPRSS2 to COVID-19, a link between SARS-CoV-2 infection outcome and other proteases such as furin and cathepsin B/L is yet to be established. A genetic variation in the proximal CTSL1 promoter, especially at position C-171A, was associated with higher blood pressure [58]. It was also demonstrated that the furin gene contributes to the pathogenesis of hypertension and is crucial to the renin–angiotensin system [55]. For instance, the coronary artery disease genetic variant rs17514846 increases furin expression and this may be related to reduced monocyte migration and proliferation in humans [56]. Accordingly, the variant rs17514846 at the 15q26.1 locus was recently linked to higher circulating monocyte chemoattractant protein-1 (MCP-1) levels and greater carotid intima-media thickness in comparison with non-carrier individuals [57]. The same study also demonstrated that furin knockdown in vascular endothelial cells reduces endothelial cell-mediated inflammation as well as monocyte adhesion to and transmigration through these cells [57]. A similar profile was observed in mice with atherosclerotic lesions treated with furin inhibitors (α-1-PDX, RP-070) [68]. Possible outcomes of COVID-19 and their associations with these genetic variations of furin and cathepsin B/L are depicted in Table 1.

### 3.2. Immune Response

Hyperinflammation is a hallmark of COVID-19. As previously mentioned, an association between the degree of inflammation resulting from SARS-CoV-2 infection and disease severity and outcome has been observed. In fact, studies have attempted to investigate the inflammatory responses of patients with different severities of COVID-19 (Table 2). Both moderate and severe patients exhibit hyperinflammation characterized by increased circulating levels of cytokines and chemokines [12,29,41,69,70,71], whereas those with mild disease display normal levels of these inflammatory mediators [12,70,71]. Patients with moderate and severe disease also present with increased levels of chemokines in their bronchoalveolar lavage fluid (BALF) samples, while elevated cytokines are only observed in the BALF of individuals with severe disease [72].

Differences in COVID-19 severity have been linked to not only inflammatory mediator release, but also to changes in the numbers of inflammatory cells (Table 2). In fact, those with mild disease present normal peripheral blood lymphocyte counts and diminished numbers of neutrophils [12,70,71]. Evidence also indicates that individuals with moderate disease display an increased accumulation of mononuclear cells in their BALF samples, whereas those with severe COVID-19 present with a higher percentage of BALF neutrophils [72]. At the systemic level, low numbers of CD4^+^ and CD8^+^ cells have been observed [12,29,41,69,70,71,72]. In a detailed analysis of leukocyte subsets, Silvin et al. [73] demonstrated that patients with severe disease exhibit an accumulation of immature neutrophils (CD10^Low^CD101^−^ and CD10^Low^CD16^Low^ cells) and a reduction of the CD14^Low^CD16^High^ monocyte population in their peripheral blood in comparison with those of individuals who were negative for SARS-CoV-2 or presented mild disease. Lower frequencies of circulating T and B lymphocytes were also noted in these patients, as well as an increase of ROS- and NO-producing monocytes. Enhanced numbers of ROS/NO-releasing cells and immature neutrophils were observed in the BALF levels of severe patients [73]. Another study identified distinct immune signatures based on peripheral blood lymphocyte counts and activation of individuals with COVID-19 and associated them with disease severity [75]. These studies indicate the importance of monitoring the profiles of leukocytes to aid in the management of SARS-CoV-2 infection.

Less common in children, a multiple organ disorder associated with hyperinflammatory shock has been observed in this age group. A report from the United Kingdom indicated that, despite being negative for COVID-19, eight children developed a severe syndrome characterized not only by respiratory distress and pneumonia, but also by hypotension, rashes, conjunctivitis, peripheral edema, generalized pain, and diarrhea [19]. Similar symptoms were observed in SARS-CoV-2-positive critically ill American children [76].

These different patterns of inflammation in COVID-19 patients may be related to differences in the expression of genes involved in the cellular and humoral responses to SARS-CoV-2 (Table 1). The expression patterns of human leukocyte antigen – DR isotype (HLA-DR) and inflammatory mediator genes are known to highly influence the immune response to infectious diseases. Nonetheless, it is not surprising that they have been investigated in COVID-19. Peripheral blood HLA-DR^+^CD8^+^ and HLA-DR^+^CD4^+^ T-cell populations were elevated in patients with mild to moderate infection in comparison with healthy subjects [27,30]. These cells were found to co-express CD38 and were larger producers of granzymes and perforin, preceding the resolution of symptoms [30]. In contrast, patients with the severe form of the disease displayed lower expression of HLA-DR on their circulating monocytes than healthy individuals, a response that was associated with increased circulating levels of IL-6 produced by both blood monocytes and CD4^+^ cells [59]. In another study, HLA binding affinity for SARS-CoV-2 proteome was assessed by in silico analysis [77]. The study showed that the HLA-B*46:01 is the allele with the smallest number of binding peptides for SARS-CoV-2 and that other alleles such as HLA-A*02:02, HLA-B*15:03, and HLA-C*12:03 were potentially the greatest SARS-CoV-2 antigen-presenting molecules. As IL-6 production downregulates HLA-DR expression on monocytes of severe patients, alterations in the individual expression of this cytokine would be also worth investigating. Interestingly, patients with severe COVID-19 exhibited higher levels of plasma IL-6 and expressed lower levels of HLA-DRA and HLA-DRB1 in their peripheral blood and BALF monocytes/macrophages than individuals who were negative to SARS-CoV-2 or those with mild disease [73].

IFN plays an essential role in the host responses to viral infections; therefore, reduced levels of this cytokine may result in a defective viral clearance and higher severity of SARS-CoV-2 infection and mortality. In an early study, Blanco-Melo et al. [74] demonstrated that severe COVID-19 patients display a hindered pulmonary and systemic production of IFNs. Furthermore, IFN-mediated responses were suggested to be delayed by SARS-CoV-2 infection [78]. Neglected systemic levels of IFN-γ were detected in a patient with mild to moderate COVID-19 [30]. The authors also investigated and detected in this patient the single-nucleotide polymorphism rs12252-C/C in the gene IFITM3, involved in the encoding of the interferon-induced transmembrane protein 3 and previously linked to increased risk to severe influenza infections [79]. The IFITM3-rs12252-C/C variant was detected in ~26% of the Chinese population, which presented a mortality rate of 6% for COVID-19. Although no other studies have analyzed IFN-γ profiles in other populations affected by SARS-CoV-2, IFITM3-rs12252-C/C was linked to a high risk of influenza in both the White and East Asian populations [60], suggesting that this variant may also be largely detected around the globe. Of note, some individuals with critical disease presented IFN in their bronchoalveolar lavage and expressed genes associated with IFN release [80]. Whether these patients are able to deal better with SARS-CoV-2 infection remains to be investigated. More recently, genetic variants of TLR3 and IRF7, which result in deficiency of such pathways, were detected in whole blood samples and linked to impaired production of IFN-α and severe COVID-19 [81]. Additionally, a significant number of individuals with life-threatening SARS-CoV-2 infection was found to present IgG auto-antibodies against IFN-α and/or IFN-ω [82]. The same study demonstrated that these molecules neutralize the ability of IFN-α to block viral infection in vitro. Of note, these auto-antibodies were not present in patients who were asymptomatic or had mild disease. Overall, these studies indicate an essential role for IFN in the host response to SARS-CoV-2. However, further studies are necessary to determine IFN definite roles in the susceptibility and resistance to COVID-19.

## 4. Conclusions

Much is yet to be understood about COVID-19. As discussed, the SARS-CoV-2 infection is a systemic and multi-factorial disease to which mortality and morbidity depend on several aspects of the host response to infection, especially on the intrinsic differences between the individuals infected with SARS-CoV-2. So far, the findings point towards an important role for the intrinsic expressions and activities of ACE2, the discussed tissue proteases, and the immune pathways involved in individuals and COVID-19 outcomes. However, research on this matter is a novel field with many questions that remain to be answered. Therefore, further studies on the mechanisms underlying an individual’s susceptibility to develop severe or fatal COVID-19 are of great need, so better management tools can be developed for SARS-CoV-2 infection.

## Figures and Tables

**Table 1:** Angiotensin-converting enzyme 2 (ACE2), tissue protease, and immune marker expression patterns involved in the severity of SARS-CoV-2 infection.

Protein	Species	Protein Expression/Activity	Variant/Polymorphism	Possible Effect	References
ACE2	Human	-	rs143936283 (E329G) and rs73635825 (S19P) allele variant	mild to moderate COVID-19	[50]
	Human	-	high allele frequency in the QTL expression quantitative trait loci variants – associated with higher ACE2 expression	mild to moderate COVID-19	[51]
	Human	increased lung expression	-	severe COVID-19	[23,52]
	Human	no expression alteration	-	mild to moderate COVID-19	[13,53]
	Human	decreased activity	-	severe COVID-19	[54]
TMPRSS2	Human	increased activity	-	severe COVID-19	[53]
Furin	Human	-	G allele of 1970C > G	severe COVID-19	[55]
	Human	increased expression	rs17514846 variant	severe COVID-19	[56,57]
CatL	Human	-	proximal CTSL1 promoter at position C-171A	severe COVID-19	[58]
HLA-DR	Human	low expression	-	severe COVID-19	[59]
IFN-γ	Human	-	rs12252-C/C in the gene IFITM3	mild to severe COVID-19	[30,60]

ACE2: angiotensin-converting enzyme 2; CatL: cathepsin L; CTSL1: cathepsin L1; HLA-DR: human leukocyte antigen – DR isotype; IFITM3: interferon-induced transmembrane protein 3; IFN-γ: interferon gamma; TMPRSS2: transmembrane protease serine 2.

**Table 2:** Immune profiles and disease outcomes in adults infected with SARS-CoV-2.

Disease Severity	Complications	Immune Response Intensity	Cellular Immune Response	Cytokine/Chemokine Responses	Disease Outcome	References
Mild	-	-	peripheral blood: normal levels of CD4^+^, CD8^+^, CD19^+^, and NK cells	normal plasma cytokine levels	-	[71]
	-	-	peripheral blood: low neutrophil counts (2.34 (1.2–2.81) × 10^9^/L) and normal lymphocyte counts	normal plasma cytokine levels	-	[70]
	-	-	peripheral blood: low neutrophil and normal lymphocyte counts	-	-	[12]
	fever, cough, diarrhea, myalgia, anosmia/ageusia	-	peripheral blood: increase of CD10^Low^CD101^+^ neutrophils, CD14^High^CD16^High^ monocytes, IFN-producing monocytes BALF: increase of monocyte/macrophage counts	low CXCL8 and increased IFN-α plasma levels	-	[73]
Moderate	pneumonia, hepatic failure	hyperinflammation	peripheral blood: normal lymphocyte counts, normal CD4^+^ and CD8^+^ cell counts	increase of plasma IL-1ra, IL-6, IL-18, CTACK, MIG, M-CSF, IL-10, IP-10, IFN-γ	recovery	[41]
	-	hyperinflammation	BALF: CD14^+^ cells, high % of plasmacytoid dendritic cells, T and B lymphocytes and NK cells	increase of BALF CXCL9, CXCL10, CXCL11, and CXCL16	recovery	[72]
	dyspnea and pneumonia	-	peripheral blood: low CD14^Low^CD16^High^ monocyte counts	increase of plasma calprotectin	-	[73]
**Severe**	pneumonia, acute respiratory distress syndrome, RNAemia, acute cardiac and kidney injury, secondary infection, shock, median systolic pressure of 145, respiratory rate > 24 breaths/min, increased pro-thrombin time and D-dimer level	hyperinflammation	peripheral blood: high numbers of neutrophils: 10.6 (5.0–11.8) × 10^9^/L, low lymphocyte counts: 0.4 (0.2–0.8) × 10^9^/L	increase of plasma levels of IL-2, IL-7, IL-10, GSCF, IP-10, MCP-1, MIP-1α, and TNFα	increased mortality	[69]
	pneumonia, acute respiratory distress syndrome, hepatic and kidney failure, cardiac failure, shock	hyperinflammation	peripheral blood: low % of neutrophils and lymphocytes, low counts of CD4^+^ (0.3 (0.2–0.4) × 10^9^/L) and CD8^+^ cells (0.1 (0.1–0.2) × 10^9^/L)	increase of plasma IL-1ra, IL-6, IL-18, CTACK, MIG, MCP-3, M-CSF, MIP-1α, HGF, IL-10, IP-10, IFN-γ	increased mortality	[41]
	acute respiratory distress syndrome	hyperinflammation	peripheral blood: normal neutrophil counts, low % of lymphocytes and eosinophils, low counts of T cells (0.5 × 10^9^/L), CD8^+^ cells (0.15 × 10^9^/L) and regulatory T cells	increase of serum IL-6, decrease of IL-8	-	[29]
	acute respiratory distress syndrome	hyperinflammation	BALF: increased % of neutrophils, reduced % of dendritic cells, presence of M1 and M2 macrophages	increase of BALF IL-8, IL-6, IL-1β, CXCL9, CXCL10, and CXCL11	increased mortality	[72]
	-	-	peripheral blood: low numbers of CD4^+^, CD8^+^, CD19^+^, and NK cells	increase of plasma IL-6	-	[71]
	acute respiratory distress syndrome	-	peripheral blood: normal neutrophils, low CD4^+^ and CD8^+^ cell counts	increase of plasma IL-6	-	[70]
	acute respiratory distress syndrome	-	peripheral blood: low lymphocyte CD8^+^ cell counts	increase of IL-6, IL-10, IL-2, and IFN-γ	-	[12]
	-	hyperinflammation	-	neglected production of IFNs, increase of lung (CCL2, CCL8, and CCL11) and systemic (CCL2, CCL8 CXCL2, CXCL8, CXCL9, and CXCL16) chemokines, and systemic IL-6 and IL-1ra	-	[74]
	acute respiratory distress syndrome	-	peripheral blood: increase of total neutrophils, and CD10^Low^CD101^−^ and CD10^Low^CD16^Low^ neutrophil counts, low numbers of CD14^Low^CD16^High^ monocytes, low frequencies of CD4^+^, CD8^+^, and CD19^+^ cells, increase of ROS- and NO-producing monocytes BALF: increase of ROS- and NO-producing monocytes/macrophages, accumulation of immature neutrophils	increase of plasma calprotectin, CXCL-8, CXCL-12, and IL-6 levels	-	[73]

BALF: bronchoalveolar lavage fluid; CTACK: cutaneous T-cell-attracting chemokine; GSCF: granulocyte-colony stimulating factor; HGF: hepatocyte growth factor; IFN-γ: interferon gamma; IL-10: interleukin 10; IL-18: interleukin 18; IL-1ra: interleukin-1 receptor antagonist; IL-2: interleukin 2; IL-6: interleukin 6; IL-7: interleukin 7; IP-10: interferon gamma-induced protein 10; MCP-1: monocyte chemoattractant protein-1; MCP-3: monocyte chemotactic protein-3; M-CSF: macrophage colony-stimulating factor; MIG: monokine induced by interferon gamma; MIP-1α: macrophage inflammatory protein-1 alpha; NK: natural killer; TNFα: tumor necrosis factor alpha.
